# Editorial: Multidisciplinary approach in health: new strategies from the perspective of education, management, culture and gender

**DOI:** 10.3389/fpsyg.2024.1418051

**Published:** 2024-05-06

**Authors:** Sagrario Gomez-Cantarino, M. Idoia Ugarte-Gurrutxaga, Carmen Solano-Ruiz, Beatriz de Oliveira Xavier

**Affiliations:** ^1^Department of Nursing, Physiotherapy and Occupational Therapy, Faculty of Physiotherapy and Nursing, University of Castilla-La Mancha, Toledo, Spain; ^2^Department of Nursing, Faculty of Nursing, University of Alicante, Alicante, Spain; ^3^Nursing School of Coimbra, Coimbra, Portugal

**Keywords:** education, management, culture, health, gender, sexuality, health habits

The training within the European Higher Education Area must promote equality and inclusion in both academic and social spheres, fostering respect toward cultural diversity and sexual orientation, and ensuring non-discriminatory access to education, while integrating a gender perspective across all university areas.

From this standpoint, within the field of Health Sciences, there is a need for training in both knowledge and skills that facilitate the development of cultural competence (Berenguel Chacón et al., 2023) and education in affective-sexual matters (Cantarino et al., 2016).

However, do educational programs include comprehensive sexual education? Scientific literature paints a rather discouraging picture in this regard, revealing significant deficiencies that need to be addressed (Cunha-Oliveira et al., 2021). There is also evidence of inadequate training in providing healthcare in culturally diverse contexts (Sharifi et al., 2019).

It is necessary to enhance new educational approaches different from conventional ones, where the focus is not only on conceptual competencies related to the discipline but also on the development of attitudes free from biases and prejudices, and behaviors that do not discriminate against people based on their cultural background or sexual orientation (Acosta-Leal et al., 2020).

Undoubtedly, the educational project will be enriched through interdisciplinary collaboration among healthcare professionals (social workers, psychologists, educators, and public health experts). In this regard, there is a proposal that examines the interaction of sex and gender psychological roles on symptoms of stress, scatter, and anxiety: Sex and gender role differences in stress, depression, and anxiety symptoms in response to the COVID-19 pandemic over time (Arcand et al.), which states that sexual and psychological gender differences contribute to heterogeneous patterns of stress and anxiety symptoms over time in response to the COVID-19 pandemic. However, training in health sciences a should include experiential learning opportunities in diverse rotations and fieldwork, allowing students to apply theoretical knowledge in real-life situations and develop empathy and teamwork skills. Therefore, mental health and healthcare are fundamental aspects that must be addressed comprehensively in the context of gender and healthcare.

The discrimination, stigma, and lack of access to culturally competent health services, including mental health, can exacerbate the challenges faced by LGBTQ+ individuals in terms of emotional and psychological wellbeing. Thus, to address these disparities, both students and healthcare professionals must recognize and address the specific mental health needs of LGBTQ+ individuals, creating safe and prejudice-free environments where they can express their concerns and receive support. This situation is addressed in: Gender and sexuality in mental health: perspectives on the rights and mental health of lesbians, gays, bisexuals, and transgender people (LGBT) in the ASEAN region (Alibudbud). Training in cultural competence and gender sensitivity is emphasized to ensure inclusive and respectful care. It is also important to promote policies that protect LGBTQ+ rights, such as laws that promote anti-discrimination and equal access to healthcare (Ugarte-Gurrutxaga, 2020).

It is important to foster critical thinking, ethical reflection, and a commitment to continuous improvement in higher education within the health sciences. Indeed, self-esteem and professional identity are crucial elements for healthcare professionals and students. In the literature, a study reveals that perceived prejudice and psychological distress can influence these aspects (Wu et al.). Stereotypes and perceived discrimination in the workplace that can undermine self-esteem and professional confidence, negatively impacting the quality of care provided, are shown. It is essential to address these challenges to promote an inclusive and healthy work environment where healthcare professionals develop a strong identity and maintain optimal mental health to provide quality care. It is also worth noting that historically, as documented by Espina-Jerez et al., early female predecessors of modern nursing faced socio-cultural difficulties solely because of their gender due to their scientific activities such as herbalism and healing, among others, being condemned and even imprisoned for these practices. Another issue related to training in health sciences is observed in the study presented by Shiningayamwe, which addresses the implementation of educational policies in Namibia and student pregnancies in rural schools. In fact, these authors propose essential ideas to reduce pregnancies and school dropout rates, reinforcing formal education. It is recognized that sex education leads to changes in behaviors and norms, as expressed by Gradellini et al., where perspectives such as students' educational level, prior knowledge, and possible reactions to sexual topics are analyzed, without forgetting religious and cultural influences. This issue is raised and continued in the literature with a question: Purity or perversion? From taboo to fact: reflections of kindergarten teachers on age-appropriate sexuality (Lehn et al.). It is concluded that sexuality constitutes a crucial educational issue requiring further exploration to overcome barriers to its addressing, with the aim of improving the preparation and competence of future healthcare professionals.

Alzain et al. recognize the value of volunteering in personal and professional development. Thus, it is evaluated how this experience fosters competencies such as intercultural communication, teamwork, and problem-solving, as well as specific technical skills in healthcare specialties.

The authors Santiago et al. present a multicenter study investigating nursing students' knowledge about sexuality, sex, and gender diversity. It is imperative that nursing curricula integrate a deep understanding of sexuality and its diversity. This will ensure adequate preparation to comprehensively and sensitively address sexual health needs.

## Author contributions

SG-C: Conceptualization, Formal analysis, Investigation, Project administration, Validation, Visualization, Writing – original draft. MU-G: Conceptualization, Formal analysis, Investigation, Project administration, Visualization, Writing – review & editing. CS-R: Data curation, Methodology, Supervision, Writing – original draft. BO: Resources, Visualization, Writing – review & editing.
